# Practical application of cure mixture model for long-term censored survivor data from a withdrawal clinical trial of patients with major depressive disorder

**DOI:** 10.1186/1471-2288-10-33

**Published:** 2010-04-23

**Authors:** Ichiro Arano, Tomoyuki Sugimoto, Toshimitsu Hamasaki, Yuko Ohno

**Affiliations:** 1 Pfizer Global Research and Development, Pfizer Japan Inc., Yoyogi 3-22-7, Shibuya 151-8589, Tokyo Japan; 2 Department of Mathematical Health Science, Osaka University Graduate School of Medicine, Yamadaoka 1-7, Suita 567-0871, Osaka, Japan; 3 Department of Biomedical Statistics, Osaka University Graduate School of Medicine, Yamadaoka 2-2, Suita 567-0871, Osaka, Japan

## Abstract

**Background:**

Survival analysis methods such as the Kaplan-Meier method, log-rank test, and Cox proportional hazards regression (Cox regression) are commonly used to analyze data from randomized withdrawal studies in patients with major depressive disorder. However, unfortunately, such common methods may be inappropriate when a long-term censored relapse-free time appears in data as the methods assume that if complete follow-up were possible for all individuals, each would eventually experience the event of interest.

**Methods:**

In this paper, to analyse data including such a long-term censored relapse-free time, we discuss a semi-parametric cure regression (Cox cure regression), which combines a logistic formulation for the probability of occurrence of an event with a Cox proportional hazards specification for the time of occurrence of the event. In specifying the treatment's effect on disease-free survival, we consider the fraction of long-term survivors and the risks associated with a relapse of the disease. In addition, we develop a tree-based method for the time to event data to identify groups of patients with differing prognoses (cure survival CART). Although analysis methods typically adapt the log-rank statistic for recursive partitioning procedures, the method applied here used a likelihood ratio (LR) test statistic from a fitting of cure survival regression assuming exponential and Weibull distributions for the latency time of relapse.

**Results:**

The method is illustrated using data from a sertraline randomized withdrawal study in patients with major depressive disorder.

**Conclusions:**

We concluded that Cox cure regression reveals facts on who may be cured, and how the treatment and other factors effect on the cured incidence and on the relapse time of uncured patients, and that cure survival CART output provides easily understandable and interpretable information, useful both in identifying groups of patients with differing prognoses and in utilizing Cox cure regression models leading to meaningful interpretations.

## Background

In a clinical study involving patients with major depressive disorder (MDD), a long-term placebo treatment is not acceptable due to an increased risk of suicide. In such a situation, a randomized withdrawal design is one of the most useful approaches for comparing the long-term efficacy and safety of a drug and a placebo. A typical randomized withdrawal study consists of an initial phase during which all patients are given open-label active treatment followed by randomization of the responders to continued double-blind treatment with either active or placebo treatment. The advantage of this study design is the reduced duration of placebo treatment and an early-escape endpoint, such as a relapse of signs or symptoms of the disease or a lack of efficacy.

Table 1 provides a summary of the design and rates of relapse in MDD patients from several recent randomized withdrawal studies [1-5]. Common features among the studies include long-term follow-up, relatively large sample size, high incidence of non-relapsed patients, and non-excessive censoring from lost to follow-up during the period when relapse events often occur. In addition, the Kaplan-Meier method and log-rank test were most often used to examine the efficacy of the test drug compared with a placebo. The results from these randomized withdrawal studies indicate that most individuals with MDD often did not experience relapse events during the treatment period. The proportion of non-relapse patients ranged from 74.0% to 91.5% in the drug groups and 58.0% to 80.5% in the placebo groups. The proportion of patients who responded favourably to treatment, showing no subsequent signs or symptoms of the disease, were considered "cured", while the remaining patients may eventually "relapse". As a result, a long-term censored relapse-free time may appear in data. In this situation, unfortunately, standard survival analysis methods such as the log-rank test and Cox proportional hazards regression (Cox regression) may be inappropriate when analyzing such long-term censored data, as they assume that if complete follow-up were possible for all individuals, each would eventually experience the event of interest.

**Table 1:** Study designs and relapse rates from several randomized withdrawal studies in patients with MDD

Source	Study objectives, design and analysis	Reported relapse rates	
Rapaport *et al*. [1]	Escitalopram continuation treatment to prevent relapse; a multi-center, placebo controlled, randomized withdrawal study; 36-week randomized treatment; Kaplan-Meier estimate and log-rank test as primary statistical analysis	Escitalopram	26.0%* (109)
		Placebo	40.0%* (116)

Keller *et al*. [2]	Long-term efficacy and tolerability of gepirone ER; a multi-center, placebo controlled, randomized withdrawal study; 40-44 weeks of randomized treatment; chi-square test; Kaplan-Meier estimate and log-rank test as Primary statistical analysis	Gepirone ER	20.6% (26/126)
		Placebo	28.2% (35/124)

Kamijima *et al.*[3]	Efficacy, safety and tolerability of sertraline in the prevention of relapse; a multicenter, placebo controlled, randomized withdrawal study; 16-week randomized treatment; Kaplan-Meier estimate and log-rank test as primary statistical analysis	Sertraline	08.5% (10/117)
		Placebo	19.5% (23/118)

Perahia *et al*. [4]	Efficacy, safety and tolerability of duloxetine in the prevention of relapse; A multi-center, placebo controlled, randomized withdrawal study; 26-week randomized treatment; Kaplan-Meier estimate, log-rank test as primary statistical analysis	Duloxetine	17.4% (23/132)
		Placebo	28.5% (39/137)

Kocsis *et al*. [5]	Long-term efficacy and safety of venlafaxine ER in preventing recurrence; a multi-center, placebo controlled, randomized withdrawal study; 12 months randomized treatment; Kaplan-Meier estimate and log-rank test as primary statistical analysis	Venlafaxine ER	23.1%* (129)
		Placebo	42.0%* (129)

* Determined by a Kaplan-Meier estimate as the number of patients with relapse was not reported in the article.

In this paper, two methods of analysis were considered: a semi-parametric cure regression (Cox cure regression) and a tree-based method, known as survival CART (Classification And Regression Trees) [6]. The two methods are generally used for the analysis of time-to-event censored data and may provide the findings different from the standard Cox regression by assuming that there are cured individuals in the data. The former method combines a logistic formulation for the probability of occurrence of an event with a proportional hazards specification for the time of occurrence of the event [7-13], so that the effects of the treatment and other factors can be interpreted separately into those on the proportion of cured patients and the failure time of uncured patients. In the latter method, termed cure survival CART, the probability of occurrence of an event with the exponential or Weibull distribution for the time of the event occurrence is modelled using binary tree-structure. Our approach was to incorporate the LR test statistic from the fitting of the cure survival regressions for recursive partitioning procedures, although the standard survival CART method adapts the method of CART paradigm using the log-rank statistic [14]. Cure survival CART was performed to supplement the results of Cox cure regression and to provide a simple and useful interpretation. These findings would provide valuable information for the future treatment of patients with MDD. This paper is structured as follows: first, we describe the models, parameter estimations, and algorithms of the two methods. We then illustrate some aspects of the two methods, using data collected in a sertraline randomized withdrawal study in patients with MDD [3], and end with a discussion.

## Methods

### Cox cure regression

#### The model

Suppose that the data (*T*_*i*_, Δ_*i*_, ***X***_*i*_, ***Z***_*i*_) are available for an individual *i *= 1,..., *n*. *T*_*i *_denotes the time to the occurrence of the event defined by min(, *U*_*i*_), where  and *U*_*i *_are the random variables of true survival and censoring, respectively. Δ_*i *_denotes the censoring indicator Δ_*i *_= *I *( = *T*_*i*_), where *I*(·) is the indicator function. ***X***_*i *_and ***Z***_*i *_are the covariate vectors related to the cured incidence and uncured survival, respectively.

Let *λ*_*i*_(·) be the hazard function for an individual *i*. Suppose that *λ*_*i *_holds for the Cox proportional hazards model if individual *i *is uncured and *λ*_*i *_has zero-hazard otherwise. Therefore, we can write

where *λ*_0_(*t*) is the baseline hazard function, ***β ***is the *p*-dimensional parameter vector corresponding to ***Z***_*i*_, and *η*_*i *_is the indicator *η*_*i *_= 1 if individual *i *eventually experiences the event (uncured) and *η*_*i *_= 0 if individual *i *never experiences the event, with the cured incidence *c*_*i *_= Pr(*η*_*i *_= 0 | ***X***_*i*_). Here we suppose that *c*_*i *_is the logistic model given by

where ***α ***is the *q*-dimensional parameter corresponding to ***X***_*i*_, which usually consists of the form (1, *X*_*i*1_,..., *X*_*iq*-1_)^T ^with the intercept term *X*_*i*0 _= 1.

#### Parameter estimation

In general, although observing *η*_*i *_= 0 of being cured is not possible, only *η*_*i *_= 1 of being uncured when Δ_*i *_= 1 is known. For this reason, a marginal type of full likelihood considered by Boag [15] is available for Cox cure regression. Then, the logarithm of the marginal full likelihood for Cox cure regression is

where , *S*_*i*_(*t*) = exp[-exp(***β***^T ^***Z***_*i*_)Λ_0_(*t*)] and ***θ ***= (***α***^T^, ***β***^T^)^T^

The estimate of (***θ***, Λ_0_) is obtained by maximizing *l*_*f *_over (***θ***, Λ_0_). This maximization is performed using the EM (Expectation-Maximization) algorithm, given a suitable stating value (***θ***^(0)^, ) of (***θ***, Λ_0_). We prepare a suitable ***θ***^(0) ^using the Monte Carlo method [7,8]. Once we have ***θ***^(0)^, a suitable  corresponding to ***θ***^(0) ^is computed by a Newton-Raphson method discussed in Sugimoto and Hamasaki [13]. Although the optimization technique based on the EM algorithm is computationally fast, it easily fails to converge depending on starting values in Cox cure regression [10]. Therefore, providing an appropriate (***θ***^(0)^, ) prudently in advance using these methods, then we go ahead to the EM algorithm.

Using the concept of Taylor [16], the EM algorithm for Cox cure regression has been developed [8-10]. The *m*-th E-step in the EM algorithm transforms *l*_*f *_into the form for the complete-data log likelihood

for a given , where ***w***^(*m*) ^is computed by current parameter values  as  and(1)

In the *m *-th M-step algorithm, it is found that a Breslow's estimate of Λ_0 _[17],

can be utilized for the maximization of . Substituting  into Λ_0 _of  leads to a form of log partial likelihood

Then, the M-step of  over (***β***, Λ_0_) is replaced by maximizing  only over ***β ***in treating Λ_0 _as a nonparametric function. Therefore, the M-step is easily performed by maximizing  and  over ***α ***and ***β***, respectively, via a Newton-Raphson method.

Now, if ***θ***^(*m*) ^= (*α*^(*m*)^, ***β***^(*m*^) are the current estimates obtained by the M-step for a given ***w***^(*m*)^, then current estimate  of Λ_0 _is written as . For the next (*m *+1) -th E-step, ***w***^(*m*) ^is updated to ***w***^(*m*+1) ^by substituting (***θ***^(*m*^, ) into (***θ***, Λ_0_) in (1). Finally, (), which provides  becomes an estimate of (***θ***, Λ_0_) in a series of the EM-iteration. From our limited experiences with real data and simulation, it is recommended that a variety of starting value ***θ***^(0) ^be used to ensure that a global maximum is found. For further discussions, please see Sugimoto and Goto [8], Peng and Dear [9], Sy and Taylor [10] and Sugimoto *et al*. [12]

### Cure survival CART

In general, tree-based methods provide classifications of patients with differing prognoses that may help clarify the association of patient characteristics and survival. In this section, a tree-based method for censored data including long-term survivors is developed, based on cure survival regression. With respect to the cost of calculations in estimating the parameters, rather than the straightforward use of the semiparametric Cox cure regression discussed in the previous section, the fitting of parametric cure regression is more reasonable, where the exponential distribution is assumed to be an underlying distribution for the latency time of relapse. However, since an assumption of exponential distribution is restrictive, the Weibull distribution is also considered. First, the model will be described and then the algorithm will be discussed.

#### The model

It is assumed that the treated patient population is composed of both cured and uncured patients. A sample consists of vectors (*T*_*i*_, Δ_*i*_, ***X***_*i*_), *i *= 1,..., *n*, where (*T*_*i*_, Δ_*i*_, ***X***_*i*_) are the same as the definitions in Cox cure regression. The full likelihood of the tree constructed in this sample can be described as

where  is a set of terminal nodes, {*i*: ***X***_*i *_∈ } is a set of individual labels which belong to node *h*, and  represents a covariate space which provides node *h*; *λ*_*h*_(*t*), *S*_*h *_(*t*), and *c*_*h *_are the hazard function, survival function, and cure rate, respectively, for the individuals in node *h*. For the Weibull model, we write  and  with unknown parameters *μ*_*h *_and *ρ*_*h*_, while the exponential case is reduced to those of *ρ *= 1. *w*_*h *_(*T*_*i*_) is the conditional probability that the *i *-th individual in node *h *will eventually belong to the uncured category given that no event has occurred by time *T*_*i*_, such that

The major area of interest was the examining of cure rates and the identification of MDD patients with different prognoses. Parameter transformations such as log{*c*_*h*_/(1 - *c*_*h*_)} = *α*_*h*_,  and  were adapted and the EM algorithm, described in the previous section, is used to estimate parameters *c*_*h*_, *μ*_*h *_and *ρ*_*h*_. For the Weibull model, it is recommended that a suitable starting point of *ρ*_*h *_is sought in advance.

#### The algorithm

The tree-based method requires the splitting, pruning, and selection of a pruned subtree to be specified [6]. Since high-dimensional data may give a complicated tree-structure, the following specifications were considered to simplify the tree structure, and the results were subsequently interpreted.

##### Splitting

Two disjointed tree-based graphs are developed by recursively splitting data into two regions. Each split is evaluated for each of the variables, and a single variable (***X***_*i*_) and the split value resulting in the greatest reduction in impurity is selected by likelihood-ratio test statistic of cure exponential regression, to best reduce error. When growing trees, the improvement measure for a split (*s*) at node *h *into left and right daughter nodes, *l *(*h*) and *r *(*h*), respectively, is measured by *R*(*s*, *h*) = *R *(*h*) - {*R*(*l *(*h*))*R *+*r *(*h*)}, where)

represents the -2 times maximum log likelihood divided by the sample size *n *(deviance residual) obtained for the data of node *ħ *= (= *h*, *l*(*h*), *r *(*h*)); ,  and  are the maximum likelihood estimates (MLEs) of *c*_*ħ*_, *μ*_*ħ *_and *ρ*_*ħ *_obtained for node *ħ*,

In the exponential case,  holds and the hazard function is simply expressed as

A split  satisfying  over *s *∈  is selected, where the set  is composed of all possible binary splitting rules considered at node *h*. The two new daughter nodes, *l *(*h*) and *r *(*h*), determined by  are generated, and the tree as a whole grows. Such a generation of nodes is performed successively at any node until a pre-specified stopping condition (e.g., the maximal number of terminal nodes or the minimal number of observations in terminal nodes) is satisfied.

##### Pruning

The "Pruning procedure" [6] determines the order how to remove superfluous sub-trees of a largest tree  to build a good fitting simple tree-based model. This process is repeated until only a small fraction of the data remains. The final tree expresses a logical rule representing an extreme outcome group.

Methods based on within-node deviance are hired for pruning. In the CART algorithm, the performance of a tree is based on the cost complexity measure, defined by

For the binary tree,  is the number of terminal nodes, and *γ *is a penalty per terminal node . A sub-tree (a tree obtained by removing branches)  (*γ*) is the smallest optimally pruned sub-tree for any penalty *γ *of the largest tree  if

where  indicates that  is a sub-tree of ; and  is the smallest optimally pruned sub-tree such that  for every optimally pruned sub-tree .

The cost complexity pruning algorithm allows the optimally pruned sub-tree for any *γ *to be obtained. This algorithm finds the sequence of optimally pruned sub-trees  by repeatedly deleting branches of the tree for which the average reduction in impurity per split in the branch is smallest, where

The pruning algorithm is necessary for finding optimal sub-trees because the number of possible sub-trees grows very rapidly as a function of tree size.

##### Selection of a pruned sub-tree

The selection of a pruned sub-tree can be based on a re-sampling technique (cross-validation) to correct for over-optimism due to split point optimization [6]. The most popular method for obtaining tree-based estimates of prediction error (or expected deviance) is *V*-fold cross-validation. The data  are divided up into *V *sets  and subsamples  of about equal size, and trees  are grown from the subsamples . For any *γ*, an optimally pruned sub-tree (*γ*) and MLEs  and  in  are obtained.

The average cross-validated deviance residual over *V *subsamples is

Let *γ*_k* _be the value of that minimizes *R*^*cv*^(*γ*) of , *k *= 0,1,2,⋯. Then a tree  corresponding to *γ*_k* _is selected.

We adopt *V *= 10 because a smaller value of *V *is preferred to *V *= *n *in the application of CART [18]. While 10-fold cross-validation is a standard method for selecting tree size, it is subject to considerable variability. Therefore, in the application to this data, we performed 500 replications of 10-fold cross-validation and then determined *γ*_k* _to minimize the average of 500 pairs of . We will be able to use the "1-SE (Standard Error)" rule [6] to choose a simpler tree, where such an SE is directly estimated by the variation of *R*^*cv *^(*γ*) with 500 replications in this application.

## Results

### Study design and major results of a sertraline randomized withdrawal study

A multicenter, placebo-controlled, randomized withdrawal study was used to evaluate the efficacy and safety of sertraline in Japanese patients with MDD [3]. Following a 1-week observational period for washout, only those patients who responded after 8 weeks of open-label sertraline treatment were randomly assigned to receive one of two subsequent 16-week double-blind treatments with sertraline or a placebo. Patients who did not respond after 8 weeks of the open-label treatment were discontinued from the study. The primary variable was a relapse of the disease during the double-blind phase, which was defined as either (i) having a score of Hamilton rating scale for depression (HAM-D) (17 items) of 18 point or greater and a clinical global impression (compared to baseline of the open-label phase) of "no-change" or "worse", during two consecutive visits, or (ii) being unable to continue treatment due to insufficient efficacy.

**Figure 1:**
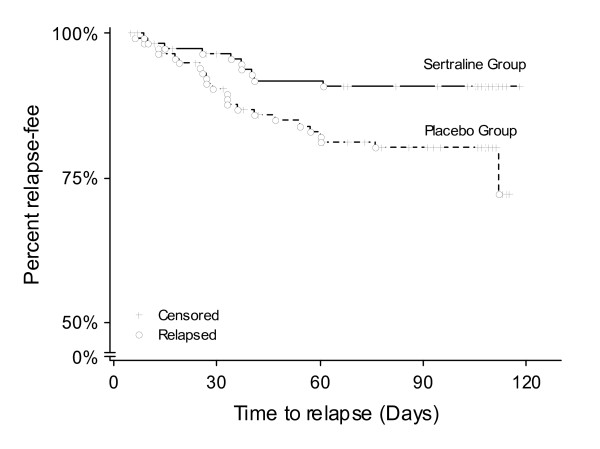
**Kaplan-Meier estimates for time to relapse in the sertraline and placebo treatment groups**.

A total of 415 patients were screened, of which 361 received sertraline in the open-label phase of the study. From this group, a total of 235 were randomized to the sertraline (*n*_*s *_= 117) or placebo (*n*_*p *_= 118) treatment groups during the double-blind phase. All analyses were based on the intent-to-treat principle. Relapse rates of 8.5% (10 of 117) and 19.5% (23 of 118) in the sertraline and placebo treatment groups, respectively were observed, which represented a statistically significant difference (Chi-squared test: p = 0.0158). Examination of the time to relapse using the Kaplan-Meier methods showed that the relapse-free rate curve for the sertraline treatment group was significantly higher than in placebo treatment group throughout the double-blind phase (log-rank test: p = 0.0261) (Figure 1). For more details, please refer to Kamijima *et al*. [3].

The standard logistic and Cox regressions were used to identify the effect of variables on the incidence of cured and uncured patients, which were not given in Kamijima *et al*. [3]. Each model included the treatment (Sertraline = 1), baseline HAM-D score at open-label phase (OP), baseline HAM-D score at double-blind phase (DP), gender (Female = 1), age, the number of episodes, duration from the first episode, duration of this episode, interval from the previous episode, and complication of MDD (complication) (Yes = 1) as covariates (Table 2). The results from both analyses suggest significant effects of treatment and gender on relapse events and time to relapse.

**Table 2:** Result of standard logistic and Cox regressions to a sertraline randomized withdrawal study in patients with MDD

			95% CI		
				
*Standard logistic regression*	Estimates	SE	Lower	Upper	p-value
Intercept	1.686	1.687	-1.621	4.992	0.3178
Treatment	0.968	0.429	0.152	1.848	0.0239
Baseline HAM-D score at OP	0.007	0.062	-0.111	0.134	0.9096
Baleline HAM-D score at DP	-0.100	0.068	-0.240	0.029	0.1443
Gender	-1.842	0.575	-3.123	-0.817	0.0014
Age	0.026	0.020	-0.013,	0.068	0.2020
The number of episodes	0.174	0.139	-0.016	0.531	0.2111
Duration from the 1st episode	-0.002	0.003	-0.008	0.005	0.5575
Duration of this episode	-0.002	0.022	-0.041	0.046	0.9296
Interval from the previous episode	0.004	0.007	-0.008	0.019	0.5553
Complication	0.572	0.413	-0.241	1.388	0.1654

Maximum log-likelihood	-81.603				
AIC	185.206				

			**95% CI**		
				
***Standard Cox regression***	**Estimates**	**SE**	**Lower**	**Upper**	**p-value**

Treatment	-0.845	0.388	-1.604	-0.086	0.0293
Baseline HAM-D score at OP	0.005	0.053	-0.099	0.109	0.9222
Baseline HAM-D score at DP	0.063	0.059	-0.052	0.180	0.2847
Gender	1.639	0.543	0.575	2.702	0.0025
Age	-0.023	0.018	-0.058	0.012	0.1977
The number of episodes	-0.152	0.130	-0.406	0.102	0.2399
Duration from the 1st episode	0.002	0.003	-0.004	0.007	0.5729
Duration of this episode	0.005	0.019	-0.033	0.042	0.8038
Interval from the previous episode	-0.003	0.006	-0.014	0.009	0.6274
Complication	-0.518	0.358	-1.221	0.184	0.1482

Maximum log-likelihood*	-180.917				
AIC	381.834				

* maximum full log-likelihood

### Application of Cox cure regression and cure survival CART to sertraline data

Cox cure regression, including influential covariates for both cured incidence and uncured survivals, provides flexibility in model building. However, this approach may open discussions on the possibility of an overparameterization of models and the identifiability between the parameters of cured incidence and uncured survivals [10], although the parameters of the standard cure model are identifiable in some sense [19]. In our situation, there was more scientific and practical interest in estimating the cured incidence as the objective of the study was to show how well the drug prevents an eventual episode of recurring illness (cured incidence), compared with placebo and, further, how other covariates influence cured incidence. Thus, in selecting the covariates, we considered that, giving priority to cured incidence over uncured survivals, a minimal number or no covariate in uncured survivals may be appropriate, and then we suggested the following guidelines on variable selection in our situation: (1) treatment is always factored into cured incidence, (2) either a minimal number or no covariates are included into uncured survivals, (3) a covariate already included into cured incidence is not included into uncured survivals. Following these guidelines, the "best" subset of all possible combinations of covariates can be selected by a minimum value of Akaike's information criterion (AIC) [20], given by  is (***θ***, Λ_0_) that maximizes *l*_*f *_(***θ***, Λ_0_), discussed in the previous Methods section.

The result of Cox cure regression after variable selection is summarized in Table 3. The value of AIC suggested the best subset of treatment and baseline HAM-D score at DP, which were included into cured incidence, and gender and complication were included into uncured survivals. For the cured incidence, the positive estimate for treatment indicated a higher cure rate for patients who received sertraline treatment, while the negative estimate for baseline HAM-D score at DP indicated a lower cured incidence for patients with a higher value of the score. In contrast, for uncured survivals, the positive estimate for gender indicated an earlier occurrence of relapse for female patients, and the negative estimate for complication indicated a prolonged occurrence of relapse for patients with any complications of MDD.

**Table 3:** Result of best subset for Cox cure regression

			95% CI		
				
*Cured incidence*	Estimates	SE	Lower	Upper	p-value
Intercept	1.571	2.870	-4.053	7.196	0.5840
Treatment	1.177	0.429	0.335	2.018	0.0061
Baseline HAM-D score at DP	-0.122	0.071	-0.262	0.018	0.0869

			**95% CI**		
				
***Uncured survivals***	**Estimates**	**SE**	**Lower**	**Upper**	**p-value**

Gender	1.953	0.535	0.905	3.001	0.0003
Complication	-0.967	0.418	-1.798	-0.159	0.0193

Maximum log-likelihood	-179.578				
AIC	369.155				

The result of best subset of covariates for standard logistic and Cox regressions with the minimum value of AIC is shown in Table 4. Standard logistic and Cox regressions suggested the effects of treatment and gender, which were in agreement with the result of Cox cure regression. In addition to the baseline HAM-D score at DP and complication, the standard logistic regression indicated a weak effect on the number of episodes. On the other hand, standard Cox regression was not able to detect the effect of baseline HAM-D score at DP, but it indicated a weak effect on the number of episodes. This was a major discrepancy between Cox cure regression and standard Cox regression. Compared with the best subset of covariates of standard logistic and Cox regressions, Cox cure regression suggested the covariates that were more important for cured incidence but less important for the uncured survivals, and vice versa. The subset for Cox cure regression provided a smaller value of AIC than that of standard Cox regression. The standard Cox regression is a special case of Cox cure regression with an infinitely small intercept in cured incidence [10]. The estimate and SE for the intercept were 1.571 and 2.870, respectively. These results support the use of Cox cure regression rather than standard Cox regression.

**Table 4:** Result of best subset for standard logistic and Cox regressions

			95% CI		
				
*Standard logistic regression*	Estimates	SE	Lower	Upper	p-value
Intercept	2.865	0.848	1.203	4.527	0.0007
Treatment	1.021	0.421	0.196	1.846	0.0153
Baseline HAM-D score at DP	-0.107	0.066	-0.236	0.222	0.1036
Gender	-1.737	0.563	-2.841	-0.633	0.0021
The number of episodes	0.133	0.114	-0.091	0.357	0.2443
Complication	0.585	0.407	-0.213	1.384	0.1507

Maximum log-likelihood	-81.868				
AIC	177.736				

			**95% CI**		
				
***Standard Cox regression***	**Estimates**	**SE**	**Lower**	**Upper**	**p-value**

Treatment	-0.913	0.379	-1.655	-0.171	0.0159
Gender	1.511	0.534	0.465	2.557	0.0046
The number of episodes	-0.131	0.109	-0.345	0.083	0.2296

Maximum log-likelihood*	-183.520				
AIC	373.040				

* maximum full log-likelihood

**Figure 2:**
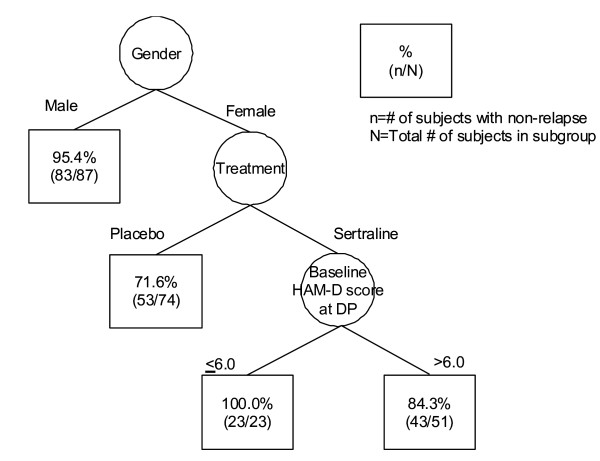
**Tree structure determined by cure survival CART analysis with exponential distribution**.

**Figure 3:**
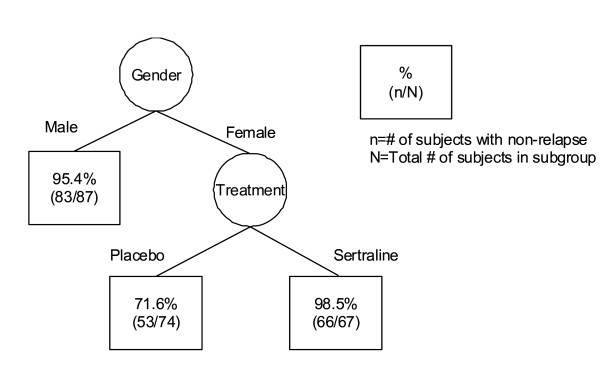
**Tree structure determined by cure survival CART analysis with Weibull distribution**.

Next, the cure survival CART was used to identify groups of patients with differing prognoses, and to refine the model previously obtained by Cox cure regression with meaningful interpretation. Four covariates (treatment, baseline HAM-D score at DP, gender and complication), which were identified by Cox cure regression, were selected to characterize the time to relapse for the sertraline data. In the application of the cure survival CART with exponential distribution, based on the average of 500 replications, the minimum cross-validated deviance residual was *R*^*cv *^(*γ*_k*_) = 1.753 (SE = 0.083), and the corresponding tree  was quite large. The 1-SE rule was used to choose a simpler tree, shown in Figure 2, which provides the cross-validated estimate of *R*^*cv *^(*γ*) = 1.791. Similarly, for the cure survival CART with Weibull distribution, the minimum cross-validated estimate based on the average of 500 replications is *R*^*cv *^(*γ*_k*_) = 1.981 (SE = 0.229), and the corresponding tree  is shown in Figure 3. As seen in Figure 2, the cure survival CART with exponential distribution showed that the primary split was gender and the secondary was treatment for the subgroup of female patients. Further partitioning of the tree was based on the baseline HAM-D score at DP of 6 points for the subgroup of female patients assigned to the sertraline treatment group. On the other hand, as seen in Figure 3, the cure survival CART with Weibull distribution provided a simpler tree structure compared with that seen in the cure survival CART with exponential distribution; there was no partitioning of the tree based on the baseline HAM-D score at DP for the subgroup of female patients assigned to the sertraline treatment group. By comparison with the cross-validated estimates between exponential and Weibull distributions, we could find that the cure survival CART with exponential distribution provided a better fit than that with Weibull distribution.

From the results obtained by the two cure survival CARTs, refined Cox cure regression was reperformed; the model included treatment, the baseline HAM-D score at DP and the interaction between the treatment and the baseline HAM-D score at DP into cured incidence, and gender and complication into uncured survivals. The baseline HAM-D score at DP was categorized into two groups: baseline HAM-D score at DP >6 ( = 1) and baseline HAM-D score at DP ≤ 6 (= 0).

**Figure 4:**
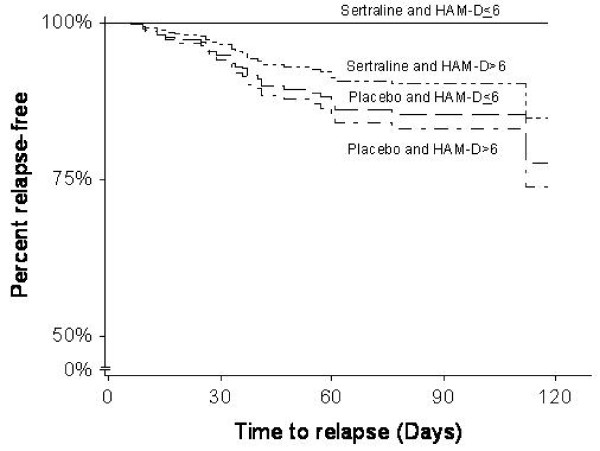
**Estimated curve for the time to relapse by refined Cox cure regression**.

The result of refined Cox cure regression is shown in Table 5. For cured incidence, a major difference between original and refined Cox cure regressions was a significant effect of the interaction between the treatment and the categorized baseline HAM-D score at DP. Refined Cox cure regression provided the negative estimates for the interaction, which indicated a higher cure rate for patients with a lower value of the score who received sertraline treatment, but a lower cure rate for patients with a higher value of the score who received the placebo. For uncured survival, there was no major difference between the original and refined Cox cure regressions. Also, as refined Cox cure regression provided a smaller AIC value than original Cox cure regression, refined Cox cure regression would lead to an improved fit. Figure 4 shows estimated curves for the time to relapse for each combination of the treatment and categorized baseline HAM-D score at DP, adjusted by gender and complication, using refined Cox regression. The important differences in cured incidence were observed between the sertraline and placebo groups with regard to the baseline HAM-D score.

**Table 5:** Result of refined Cox cure regression

			95% CI		
				
*Cured incidence*	Estimates	SE	Lower	Upper	p-value
Intercept	0.753	1.374	-1.940	3.447	0.5835
Treatment	30.954	10.001	11.353	50.555	0.0020
Baseline HAM-D score at DP	-0.233	0.646	-1.449	1.033	0.7183
Treatment × Baseline HAM-D score at DP	-30.180	13.497	-56.634	-3.725	0.0254

			**95% CI**		
				
***Uncured survivals***	**Estimates**	**SE**	**Lower**	**Upper**	**p-value**

Gender	1.926	0.535	0.878	2.974	0.0003
Complication	-0.895	0.469	-1.814	0.024	0.0563

Maximum log-likelihood	-177.499				
AIC	366.998				

## Discussion

The results obtained by Cox cure regression and cure exponential CART agree with findings reported by several authors [21-23]. For example, Nierenberg *et al*. [23] reported that a greater number of residual symptoms and higher HAM-D scores are associated with a higher probability of relapse. Although there are several inconsistencies regarding the gender difference in the course of the relapse, Kuehner [21] reported that female patients have a higher risk of earlier occurrence of relapse. In addition, co-morbidity of MMD with other illnesses has been widely reported [22]. The variable of complication used in the analysis was a binary variable of "yes" or "no", but original data included more detailed information on the number and type of complications for each patient. Therefore, further investigation on the effect of the number and type of complications on uncured survivals (or cured incidence) is necessary. Furthermore, the cut-off point for the baseline HAM-D score at DP at 6 points suggested by cure survival CART was nearly equal to the HAM-D definition of full remission (a score of 7 points or less). It is generally considered that the final goal of treatment for MDD is to achieve and maintain remission and the prevention of relapse.

Cox cure regression (or survival cure CART) generally requires a long-term follow-up [10,24]. The sertraline study discussed in this paper was intended to contribute to a new drug application in Japan, so the length of the follow-up period (16 weeks) in the study was minimized to merely detect the drug's effect, in order to reduce the duration of unnecessary exposure of patients to the drug or placebo. However, in the application of Cox cure regression to real data, before the formal analysis, an assessment of whether or not the length of follow-up is sufficient would be useful for interpreting the result. To confirm this for the sertraline data, the *q*_*n*_-test discussed by Maller and Zhou [24] was performed for the sertraline and placebo groups, constructed by the estimated cure rates and censoring distribution for this data. For the sertraline group, the observed value of 0.08547 of *q*_*n *_was between 94% and 96% critical points of the test, which supported that the length of follow-up for the sertraline group was acceptably minimal and the data had levelled off. On the other hand, for the placebo group, the observed value of 0.0085 of *q*_*n *_was much smaller than the value of 0.068 for the 95% point, which did not support that the length of follow-up for the placebo group was sufficient and that the data had levelled off. According to the results of two *q*_*n*_-tests, we could conclude that the length of follow-up period in the sertraline study was sufficient at least to detect the drug's effect compared with placebo.

In variable selection for the fitting of Cox cure regression to the sertraline data, we gave priority to cured incidence over uncured survivals. However, if there was more scientific interest in when the illness may recur rather than in the eventual cure, giving priority to uncured survivals over cured incidence could be appropriate. There are several aspects of variable selection, depending on the applications of interest.

In the paper, we discussed the two methods of cure Cox regression and cure survival CART. As described in Method section, the former method is a semiparametric regression, but the latter method use a parametric cure regression. Although the cure survival CART output provided information in refining Cox cure regression leading to meaningful interpretations for the sertraline data, note that there is the potential inconsistency between the two regressions when they consider both for data as the estimates from the semiparametric Cox cure regression and those from the parametric Cox cure regression are usually sensitive to the baseline distribution assumption. Our future challenge is to develop the semiparametric cure survival CART with fewer amounts of computations.

## Conclusions

In this study, a semi-parametric cure regression was used to investigate the latency time of recurrence observed in a sertraline randomized withdrawal study in patients with MDD. In specifying the treatment's effect on disease-free survival, account was taken of the fraction of long-term survivors and the risks associated with the relapse of the disease. In addition, a tree-based method, i.e., the cure survival CART, was used to analyze the time to event data in order to identify groups of patients with differing prognoses. The following are the main findings: (1) Cox cure regression reveals facts on who may be cured, and how the treatment and other factors effect on the cured incidence and on the relapse time of uncured patients. (2) Cure survival CART output provides easily understandable and interpretable information, useful both in identifying groups of patients with differing prognoses and in utilizing Cox cure regression leading to meaningful interpretations.

The methods discussed in this paper could be applied to the development of stratification schemes for future clinical studies and the identification of patients suitable for studies involving therapy targeted at a specific prognostic group. This is would be beneficial as it is often desirable to understand the correlation between a patient's characteristics and relapse times to aid in the design of clinical studies.

## Abbreviations

CART: Classification and regression trees; CI: Confidence interval; Double-blind phase: DP; EM: Expectation-Maximization; HAM-D: Hamilton rating scale for depression; Likelihood ratio: LR; MDD: Major depressive disorder; MLE: Maximum likelihood estimate; Open-label phase: OP; SE: Standard error.

## Competing interests

Tomoyuki Sugimoto and Yuko Ohno declare no competing interests. Ichiro Arano is an employee of Pfizer Japan, and Toshimitsu Hamasaki was an employee of Pfizer Japan between 1997 and 2004.

## Authors' contributions

ST developed the methods and FORTRAN programs to perform the methods. IA performed the analyses supervised by TS, TH and YO. IA and TH wrote the paper with contributions from ST. All authors read and approved the final manuscript.

## Pre-publication history

The pre-publication history for this paper can be accessed here:

http://www.biomedcentral.com/1471-2288/10/33/prepub
